# Poly(ethylene-co-vinyl alcohol)/Titanium Dioxide Nanocomposite: Preparation and Characterization of Properties for Potential Use in Bone Tissue Engineering

**DOI:** 10.3390/ijms23073449

**Published:** 2022-03-22

**Authors:** Waseem Sharaf Saeed, Dalal H. Alotaibi, Abdel-Basit Al-Odayni, Ahmed S. Haidyrah, Ahmad Abdulaziz Al-Owais, Rawaiz Khan, Merry Angelyn Tan De Vera, Ali Alrahlah, Taieb Aouak

**Affiliations:** 1 Engineer Abdullah Bugshan Research Chair for Dental and Oral Rehabilitation, College of Dentistry, King Saud University, Riyadh 11545, Saudi Arabia; wsaeed@ksu.edu.sa (W.S.S.); aalodayni@ksu.edu.sa (A.-B.A.-O.); krawaiz@ksu.edu.sa (R.K.); 2 Department of Periodontics and Community Dentistry, College of Dentistry, King Saud University, Riyadh 11545, Saudi Arabia; dalalotaibi@ksu.edu.sa; 3 General Administration of Research & Development Laboratories (GARDL), King Abdulaziz City for Science and Technology (KACST), Riyadh 11442, Saudi Arabia; ahydrah@kacst.edu.sa; 4 Chemistry Department, College of Science, King Saud University, P.O. Box 2455, Riyadh 11451, Saudi Arabia; aowais@ksu.edu.sa; 5 Research Center, College of Dentistry, King Saud University, Riyadh 11451, Saudi Arabia; mtandevera@ksu.edu.sa; 6 Restorative Dental Sciences Department, College of Dentistry, King Saud University, Riyadh 11545, Saudi Arabia

**Keywords:** poly(ethylene-co-vinyl alcohol), titanium dioxide nanoparticles, scaffold, TiO_2_ nanoparticle distribution, interconnection of pores, human gingival fibroblast cell activity

## Abstract

A series of poly(ethylene-co-vinyl alcohol)/titanium dioxide (PEVAL/TiO_2_) nanocomposites containing 1, 2, 3, 4 and 5 wt% TiO_2_ were prepared by the solvent casting method. These prepared hybrid materials were characterized by Fourier-transform infrared (FT-IR), X-ray diffraction (XRD), differential scanning calorimetry (DSC), thermogravimetric analysis (TGA) and scanning electron microscopy (SEM). The pores and their interconnections inside these nanocomposites were created using naphthalene microparticles used as a porogen after having been extracted by sublimation under a high vacuum at temperatures slightly below the glass transition temperature. A cellular activity test of these hybrid materials was performed on human gingival fibroblast cells (HGFs) in accordance with ISO 10993-5 and ISO 10993-12 standards. The bioviability (cell viability) of HGFs was evaluated after 1, 4 and 7 days using Alamar Blue^®^. The results were increased cell activity throughout the different culture times and a significant increase in cell activity in all samples from Day 1 to Day 7, and all systems tested showed significantly higher cell viability than the control group on Day 7 (*p* < 0.002). The adhesion of HGFs to the scaffolds studied by SEM showed that HGFs were successfully cultured on all types of scaffolds.

## 1. Introduction

Recently, researchers from different fields have devoted much effort to developing new polymeric materials that are capable of solving many problems in biomedicine and, more specifically, in tissue engineering.

Tissue engineering materials utilized in the biomedical domain are prepared from natural or synthetic polymers. Bone tissue engineering (BTE) is a strategy used to regenerate newly damaged bone tissue by combining biodegradable and biocompatible polymers as a scaffold with cells and growth factors [1]. An appropriate surface-to-volume ratio of the scaffolds promotes cell adhesion, migration and growth [1,2]. For example, an ideal scaffold candidate for BTE should have a proper structure morphology in terms of the pore density (50–90%) and pore size (>100 μm) to allow for the cell penetration, tissue growth, vascularization and sterilizability, without loss of bioactivity [2,3]. Scaffolds with an appropriate size (100–700 µm) [4,5,6] and that were interconnected lead to cell growth, uniform distribution and sufficient vascularization, while pores that are too small can lead to occlusion, preventing cell survivability and thus failure to regenerate the desired tissue [7,8].

Recently, titanium dioxide (TiO_2_) particles, also called titania particles, have attracted the attention of many researchers in the biomedical field due to their unique properties such as their relatively low price, chemical inertness, low toxicity, good stability, high refractive index, hydrophilicity, UV absorbance and excellent transparency to visible light. This metal oxide is well known to humans, allowing its use as an additive in cosmetics, pills and toothpaste due to its ability to impart whiteness and opacity to various products [9]. In addition, titania can eliminate dead cells, returning CO_2_, thus opening a way for the self-regeneration of the system [10,11,12]. These characteristics allow a wide range of applications. Due to these properties, titanium is suitable for use in medical implants. Specially designed titanium oxide nanoparticles with controlled porosity and composition are ideal for protein adsorption and enhance the tissue fixation of implants. The deposition of titanium oxide nanoparticles in the form of a film on medical implants facilitates the adhesion of the implant to the surrounding tissue. The bone-binding characteristic of TiO_2_ is correlated with the existence of surface hydroxyl groups, which can be enhanced by incorporating doping agents such as Ca, Mg, and F into titanium oxide [13]. Titania nanoparticles have been shown to possess promising antibacterial, antifungal and anticancer activities. Titanium dioxide nanoparticles have been incorporated into various polymer patches. These patches showed good antibacterial activity against Gram-positive and Gram-negative bacteria [14]. Decoration of curcumin with titanium oxide nanoparticles was effective in wound healing [15].

Interestingly, incorporating titanium oxide and curcumin into polymer patches showed good antibacterial activity against Gram-positive and Gram-negative bacteria [15]. The abovementioned characteristics of titanium dioxide make it an ideal candidate as an inorganic component for polymer–titanium oxide nanocomposites [16]. The TiO_2_ nanoparticles’ adhesion affects the biocidal action of the nanocomposites and modifies cell viability and bacteria aggregation. TiO_2_ nanoparticles are also projected as reinforcement in polymer matrices to enhance nanocomposites’ mechanical properties [17,18,19,20,21].

Nanocomposite materials involving metal oxide nanoparticles and polymers have recently attracted much attention in both the industrial and biomedical fields. The addition of a small amount of nanomaterial could improve the performance of polymeric materials because of their small size, large specific surface area and solid interfacial interactions [22,23]. Scaffolds involving nanocomposites based on TiO_2_ and polymers have also been the subject of several publications in the biomedical field. Indeed, Pelaseyed et al. [24] successfully fabricated scaffold biomaterials with a large pore size and high porosity levels from poly(lactide-co-glycolide) (PLGA) and TiO_2_ nanoparticles using an air–liquid foaming method at 20–23 °C and a relatively low pressure (2–4 bar) and a short soaking time (5 h). Among the results obtained, it was revealed that the incorporation of 10 wt% of TiO_2_ nanoparticles in the PLGA matrix showed a high antibacterial effect against strains of *Escherichia coli ATCC 8739*, demonstrated by a 100% reduction in bacterial growth in 24 h. In addition, these authors also reported that a cell adhesion assay of MG 63 cells showed that this hybrid nanomaterial had excellent cell attachment. Fibrous degradable poly(urethane ester) urea (PEUU) scaffolds reinforced with TiO_2_ nanoparticles were fabricated by Zhu et al. [23]. To increase the interfacial interaction between the PEUU and the TiO_2_ nanoparticles, these authors grafted poly(ester urethane) (PEU) on the TiO_2_. Scaffolds fabricated using the electrospinning technique had a fiber diameter of <1 μm. Images of the surface morphology of PEU-modified TiO_2_ taken by SEM and EDX revealed that this hybrid material was much more evenly distributed in the fibers. PEU-modified TiO_2_ significantly increased Young’s modulus and the tensile stress of PEUU scaffolds. Biomineralization occurred on the fibers after incubation in the simulated body fluid over 8 weeks. The PEUU scaffold with PEU-modified TiO_2_ demonstrated significantly higher cell proliferation than the pure PEUU scaffold and the PEUU scaffold with unmodified TiO_2_.

Poly(vinylalcohol-co-ethylene) (PEVAL) is a randomic and semicrystalline copolymer over the whole range of composition despite the inconsistency and nonstereospecificity of vinyl alcohol units dispensed in the copolymer chain [25]. It is well known that PEVAL is a biocompatible [26] and biodegradable polymer that could be eliminated from the human body into small and nontoxic molecules after its implantation through a fast decomposition process [27,28,29,30].

A PEVAL membrane has exceptional blood compatibility [31,32]. Indeed, the adsorbed proteins are denatured, and the cellular components of blood are activated and easily adhere to the membrane. Such a membrane inhibits the adsorption of plasma proteins, thereby inhibiting the body’s response to this material [33,34,35,36,37,38]. The effects of PEVAL membranes on blood have been evaluated in vitro separately by Nakano [37], and the results revealed that this membrane had little effect on platelets and the coagulation system. Platelets are activated when blood comes into contact with a dialysis membrane. Itoh et al. [33] applied cell ELISA to evaluate the P-selectin expressed on the platelets, and the results revealed that there was a correlation between platelet adhesion and the induction of P-selectin expression. The platelet adhesion and P-selectin expression associated with an PEVAL membrane were lower than those associated with a polystyrene (PS) membrane or a poly(methyl methacrylate) (PMMA) membrane. When neutrophils isolated from peripheral blood were stained with a hydrogen peroxide (H_2_O_2_)-sensitive fluorescent dye (dichlorodihydrofluorescein diacetate, DCFH-DA) and brought into contact with a PS or PMMA membrane in the presence of platelets, they were shown to produce reactive oxygen species (ROS) (fluorescence). In contrast, ROS were not induced when neutrophils contacted the PEVAL membrane.

PEVAL membranes are biocompatible, and exhibit specific solute removal properties and the ability to ensure material transfer in the patient’s body. In a crossover study of 12 patients [37], a study compared between the solute removal properties of a PEVAL membrane and those of a reformed cellulose (RC) membrane. The results obtained revealed that the urea clearance (K) and the Kt/V associated with the PEVAL membrane were significantly lower than those of the RC membrane. Conversely, the clearance level as estimated by Barth [39] was higher and the hematocrit level was significantly lower when the membrane was PEVAL.

In a crossover study of 11 dialysis patients, Sato et al. [36] used a PEVAL membrane, a vitamin E-linked cellulose (VE-C) membrane and a PS membrane, and they evaluated transcutaneous oxygen (TcPO_2_) at the dorsum of the foot and the fluidity of blood during dialysis. They found that the use of the PEVAL membrane moderated the drop in TcPO_2_ during initial dialysis compared with the drop associated with the use of the PS membrane and the VE-C membrane. They also indicated that the PEVAL membrane was less likely to produce disturbances in peripheral circulation. In addition, a decrease in blood fluidity was not noted with the PEVAL membrane.

The biodegradability of polymers mean that they are applied in various biomedical fields, such as tissue engineering scaffolds, artificial implants, wound dressings and carriers in drug delivery systems [40,41,42,43]. This copolymer is usually synthesized by polymerizing ethylene with vinyl-acetate to produce the poly(ethylene-co-vinyl acetate), followed by hydrolysis of the functional groups [44].

Despite the in-depth efforts by several researchers in the biomedical field, the issue of controlling infections regarding biomedical materials exists mainly in dentistry because of the presence of bacteria and other microorganisms, which form biofilms on the surface of the biomaterials. Thus, antimicrobial activity, among other properties, is the main reason for applying nanomaterials in dentistry [45,46].

In this present work, a series of nanocomposites involving PEVAL and TiO_2_ nanoparticles (PEVAL/TiO_2_) containing different TiO_2_ contents were prepared by the solvent casting route for potential application in tissue engineering in the biomedical field. Naphthalene microparticles were used as porogen removed from the hybrid material by sublimation under reduced pressure. The dispersion of TiO_2_ in the PEVAL matrix and the interactions between the two components in the hybrid material were examined by differential scanning calorimetry (DSC), X-ray diffraction (XRD) and Fourier transform infrared (FTIR). The examination of the thermal stability of the prepared material was carried out through thermogravimetry (TGA). The surface and cross-section morphologies of the PEVAL/TiO_2_ samples were examined by scanning electron microscopy (SEM). Viability and cell activity experiments on the prepared scaffolds were performed according to ISO 10993-5 and ISO 10993-12 standards. The bioviability (cell viability) of human gingival fibroblasts (HGFs) was evaluated after 1, 4 and 7 days using Alamar Blue^®^.

## 2. Material and Methods

### 2.1. Chemicals

Poly(ethylene-co-vinyl alcohol) (PEVAL) (ethylene, 32% mol; $M_{n}=2\times 10^{4}\;g\cdot {\mathrm{mol}}^{-1}$), titanium (IV) oxide nanopowder (primary particle size, 21 nm) (TiO_2_) and naphthalene beads (purity, >99%) (Naph) were purchased from Sigma Aldrich (Taufkirchen, Germany). The isopropyl alcohol (purity, 99.0%) used as solvent was supplied by WINLAB Chemical, UK. All chemicals were used without further purification.

### 2.2. Preparation of the PEVAL/TiO_2_ Nanocomposite by the Solvent Casting Method

The PEVAL/TiO_2_ nanocomposite was prepared by the solvent casting method. In a 100-mL flask, 1.0 g of PEVAL was completely dissolved in 10 mL of equal volumes of isopropyl alcohol and water at 75 °C under vigorous stirring until complete dissolution of the polymer. Next, a known amount of TiO_2_ nanoparticles was dispersed in the PEVAL solution under continuous stirring for 1.0 h and then sonicated for 30 min to prevent agglomeration of the nanoparticles. The final PEVAL/TiO_2_ suspension was then cast in a Teflon petri dish, air bubbles were removed by shaking and blowing air, and the mixture was dried at ambient temperature for 24 h followed by 24 h at 50 °C in a vacuum oven to remove the solvent traces altogether. A series of PEVAL/TiO_2_ nanocomposites containing 1, 2, 3, 4, and 5 wt% TiO_2_ were prepared by the same procedure under the conditions summarized in Table 1.

### 2.3. Preparation of PEVAL/TiO_2_ with Interconnected Pores

**Scheme 1:**
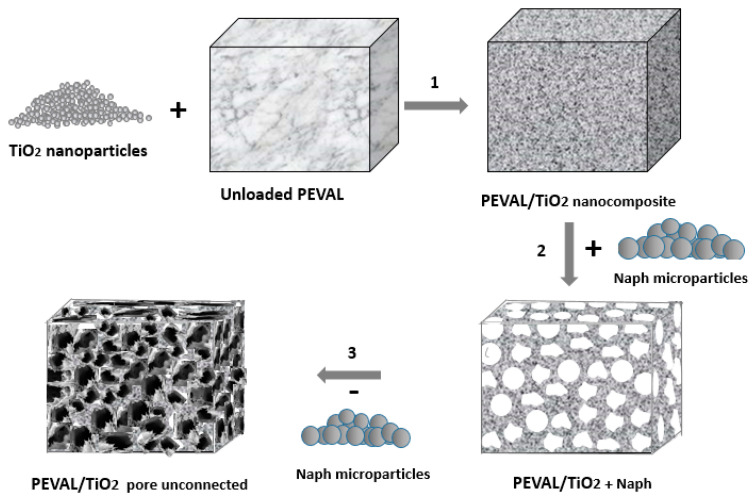
Hypothetical scheme depicting the preparation process of interconnected poly(ethylene-co-vinyl alcohol)/titanium dioxide using naphthalene. (1) Incorporation of TiO_2_ nanoparticles into the PEVAL matrix; (2) incorporation of Naph microparticles in the PEVAL/TiO_2_ nanocomposite; (3) removing the Naph microparticles by sublimation.

The PEVAL/TiO_2_ hybrid materials containing interconnected pores were prepared as described in Scheme 1. A known amount of PEVAL/TiO_2_ hybrid material was dissolved in an equal volume ratio of isopropyl alcohol and water at 75 °C. Many naphthalene microparticles with sizes between 100 and 250 μm were used as porogens, representing 60% by weight of the total mass (Naph + nanocomposite). These were added to the mixture to obtain a highly viscous mixture. The resulting suspension was placed in an ultrasonic bath for about 30 min using a degassed heating ultrasonic bath and then immediately dried under a vacuum. The films were then prepared by smoothly casting the polymeric solutions over a perfect horizontal Teflon plate surface obtained using a spirit level. The extraction of the porogen from the polymeric material was realized after solvent evaporation by sublimation under reduced pressure using a vacuum oven maintained at 60 °C for 24 h. The absence of any traces of solvent or naphthalene in the prepared material was proved by DSC analysis through the total disappearance of the endothermic peak corresponding to the evaporation of the solvent and the fusion of naphthalene.

### 2.4. Characterization

#### 2.4.1. FTIR Analysis

The FTIR spectra of all samples were recorded by a Nicolet 6700 FT-IR, Thermo Scientific (Waltham, MA, USA) at 25 °C. At least 32 scans, with an accuracy of 2 cm^−1^, in all cases were signal-averaged. The samples were dried under a vacuum at 40 °C and were analyzed as thin films, except for the TiO_2_ nanoparticles examined in powder form.

#### 2.4.2. DSC Analysis

The DSC thermograms of PEVAL/TiO_2_ nanocomposites and their components were obtained using a Shimadzu DSC 60A (Shimadzu, Kyoto, Japan). Samples weighing between 8 and 10 mg were packed in aluminum pans before being placed in the DSC cell, then they were scanned from −50 to +250 °C under nitrogen atmosphere gas with a heating rate of 20 °C·min^−1^. All the thermograms were collected from the second scan run. The glass transition temperature, T_g_, was accurately deduced from the inflection point of the thermal curve as the midpoint in the variation in the heat capacity with temperature, and the melting point, T_m_, was taken from the top of the endothermic peak.

#### 2.4.3. Thermogravimetric Analysis

A TGA of virgin PEVAL and PEVAL/TiO_2_ hybrid materials was performed under dynamic nitrogen gas on a TGA/DSC1 Mettler–Toledo thermogravimeter (Columbus, OH, USA). Samples weighing between 10 and 14 mg were loaded into the TGA aluminum pan then heated from 25 to 800 °C at a heating rate of 20 °C·min^−1^.

#### 2.4.4. XRD Analysis

The crystalline structures of all samples were examined using XRD analysis on an X-ray diffractometer (RigakuDmax 2000, The Woodlands, TX, USA) using a Cu anode tube, a tube voltage of 40 KV/40 mA and generator current of 100 mA. All specimens were examined at 2θ = 5–60° at a scanning rate of 1.0° min^−1^.

#### 2.4.5. SEM Analysis

SEM micrographs of the hybrid materials’ surface and cross-section morphologies before and after pore interconnection were obtained by a FESEM (JEOL-JSM-2100F) scanning electronic microscope at an accelerating voltage of 10 kV. Samples in the form of a thin disc were first sputter-coated with a thin layer of gold and then observed at a magnification range of 350–200×.

#### 2.4.6. Porosity and Pore Size Distribution

The porosity of the prepared scaffolds was determined using a pycnometer using n-decane (a nonsolvent for PEVAL) as a displacement fluid as described in the literature [47]. The pore diameter and pore volume distributions of the prepared hybrid materials were determined using a Quartachrome mercury intrusion porosimeter, Poremaster 60, FL (London, UK).

#### 2.4.7. SEM of Seeded Scaffolds

SEM was performed to assess HGF binding and morphology on the examined scaffolds. Scaffolds were seeded with 5 × 10^4^ cells/scaffold and cultured for 1, 4 and 7 days. At these time points, the constructs were rinsed in a 0.1 M sodium cacodylate buffer and fixed with 3% glutaraldehyde in a cacodylate buffer for 30 min. The samples were then treated with 2% osmium tetroxide for 2 h. After being rinsed with a sodium cacodylate buffer, the constructs were then dehydrated through a series of graded ethanol solutions: 75, 95, and 100% *v*/*v*, and 100% *v*/*v* dried over anhydrous copper sulfate for 15 min. The constructs were then critically dried using a hexamethyldisilazane/ethanol mixture (50/50 by weight) followed by 100% hexamethyldisilazane for 20 min. The samples were air-dried overnight in a fume hood before being mounted on aluminum stubs using carbon tabs. The samples were coated with gold by an automatic coater for 4 min to achieve a conductive layer thickness of approximately 200 nm. Scanning electron micrographs were taken using a Philips XL-20 scanning electron microscope at 20 kV.

#### 2.4.8. Cellular Activity

PEVAL/TiO_2_ scaffolds containing 0, 1, 2, 3, 4 and 5 wt% of TiO_2_ filler were cut into 1 × 1 cm pieces then sterilized under UV light for 30 min. Human gingival fibroblasts (HGF) were cultured in 24-well plates, seeded at 5 × 10^4^ cells/well using HGF basal medium). After 24 h of incubation, the scaffolds were placed on the monolayer and incubated at 37°C (5% CO_2_/95% air) for 7 days using a negative control comprising only cells. This experiment was performed following ISO 10993-5 and ISO 10993-12 standards. The bioviability (cell viability) of HGFs was evaluated after 1, 4 and 7 days using Alamar Blue^®^. Although a linear relationship between fluorescence and cell number has been established, the level of fluorescence can be affected by both an alteration in cell number (proliferation) and/or cell activity [48]. Therefore, at the required time points (Day 1, Day 4 and Day 7), the medium was removed and a fresh HGF basal medium containing 10% *v*/*v* Alamar Blue^®^ was added to each well according to the manufacturer’s instructions. After 1 h of incubation at 37 °C, 200 μL samples from each well, in triplicate, were placed in individual wells of a 96-well plate. Fluorescence intensity was measured with a plate reader (Tecan fluorescent lamp) using an excitation wavelength of 540 nm and an emission wavelength of 570 nm. The fluorescence readings of the samples without cells (i.e., the negative control) were determined to detect any dye changes occurring in the absence of cells. These negative controls did not indicate any significant interaction between Alamar Blue^®^ and the composites. Hence, the fluorescence was corrected using the value of a 10% solution of Alamar Blue^®^ in a medium without cell seeding in the presence of the composite discs as a negative control. The experiments were carried out in triplicate and repeated twice.

## 3. Results and Discussion

### 3.1. FTIR Analysis

**Figure 1:**
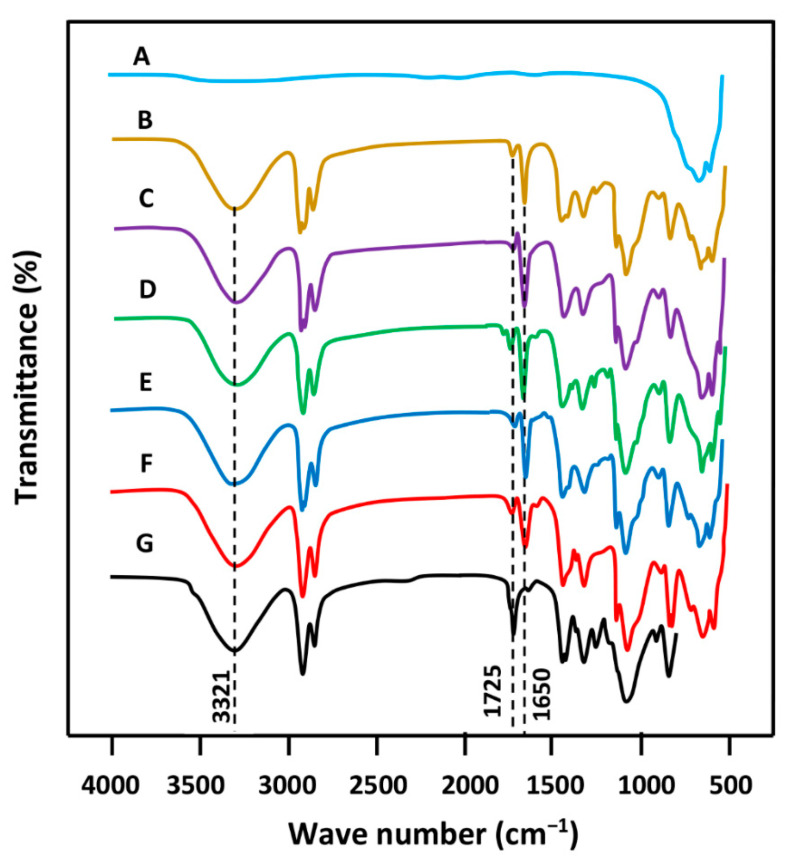
FTIR spectra of: (A) TiO_2_ nanoparticles, (B) PEVAL/TiO_2_-5, (C) PEVAL/TiO_2_-4, (D) PEVAL/TiO_2_-3, (E) PEVAL/TiO_2_-2, (F) PEVAL/TiO_2_-1and (G) pure PEVAL.

Figure 1 shows a comparison between the FTIR spectra of the TiO_2_/PEVAL nanocomposites with those of their pure components. A comparison of the absorption spectra of the different groups of PEVAL in the composites with those of the virgin copolymer reveals a significant increase in signal strength at 1650 cm^−1^ and a simultaneous decrease in the band at 1725 cm^−1^. The first absorption band is attributed to the bending modes of reflection in the plane of the hydroxyl bond attributed to water absorption [49,50], and the second band to the carbonyl group of residual acetate substituent. The simultaneous increase in the intensity of the OH absorption band and the decrease in that of the carbonyls can be reflected in the hydrolysis of the residual acetate units due to the presence of TiO_2_ nanoparticles. The absorption bands localized at 1138 and 1083 cm^−1^ are assigned to the stretching of the C-O of the crystalline sequences of the alcoholic units and the stretching of the C=O and the bending of the OH of the amorphous sequences of the copolymer [50]. The broad absorption band of the hydroxyl stretching groups of the vinylic units of the copolymer and of the water from the moisture, centered at 3321 cm^−1^, did not show any significant change.

### 3.2. XRD Analysis

**Figure 2:**
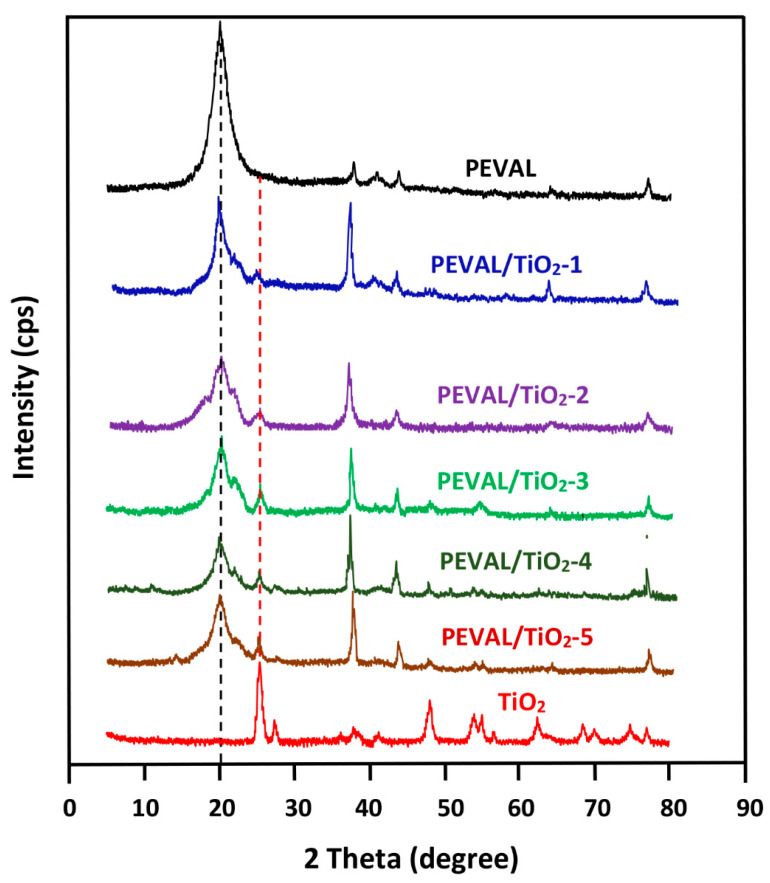
The XRD patterns of TiO_2_ nanoparticles, pure PEVAL and their nanocomposites with different compositions.

The XRD patterns of the TiO_2_ nanoparticle powder, PEVAL and these nanocomposites with different compositions are grouped in Figure 2. The spectrum of pure TiO_2_ shows the signals characterizing the anatase form of this compound (JCPDS-21-1272), in which the main ones are observed at the 25.0 (101), 37.5 (004), 48.0 (200), 54 (105), 55 (211), 62.5 (204), 69.0 (116), 70 (220) and 75.0 (215) crystal planes [51]. However, the XRD pattern of neat PEVAL presents an ultimate broad and intense peak at 20.0°, characterizing the crystalline fraction of this copolymer [52]. However, as can be seen in the spectra of the nanocomposites, the incorporation of TiO_2_ into PEVAL shows practically no change either in the crystalline structure of the copolymer or in that of the nanofiller. This indicates that the TiO_2_ nanoparticles are found in the PEVAL matrix in aggregates encrusted in its amorphous part.

### 3.3. DSC Analysis

**Figure 3:**
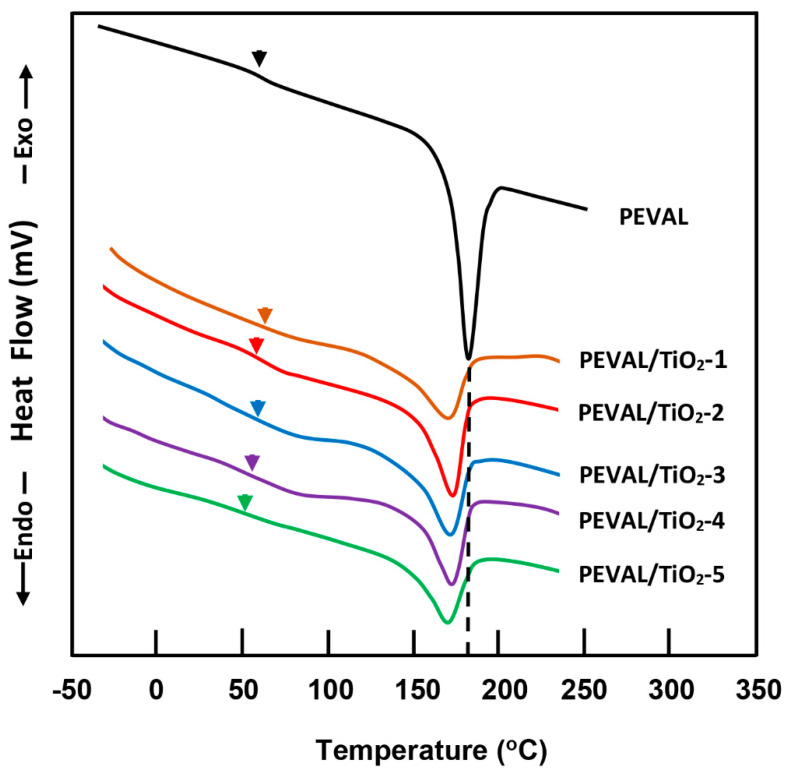
DSC thermograms of the virgin PEVAL and PEVAL/TiO_2_ nanocomposites with different TiO_2_ contents.

The DSC thermograms of PEVAL and PEVAL/TiO_2_ nanocomposites are grouped in Figure 3 and the glass transitions and melting temperatures deducted are gathered in Table 2. The thermal curve of neat PEVAL indicated 60, 184 °C and 66.78 J∙g^−1^ for the glass transition, the melting temperatures and the melting enthalpy, respectively, which agree with the literature regarding the same ethylene/vinyl alcohol composition [53]. However, for the PEVAL/TiO_2_ hybrid materials, these two properties shifted towards low temperatures, particularly the melting temperature (from 183 to 170 °C), which was also accompanied by a broadening of the melting peaks and a significant decrease in the heat of fusion of PEVAL. This reveals that the incorporation of TiO_2_ nanoparticles even in small quantities into the copolymer matrix considerably affected the thermal properties of PEVAL. This phenomenon is mainly due to the uniform dispersion of the TiO_2_ nanoparticles in the two copolymer structures (amorphous and crystalline). This increased the free volume between the polymer chains, leading to a decrease in the intensity of the interaction forces, mainly due to the hydrogen bonds between the chains of the PEVAL, leading to a decrease in its crystallinity, thus favoring the sliding of the chains on one another. Such phenomena have also been observed by other researchers studying similar nanocomposites [54,55].

### 3.4. TGA

**Figure 4:**
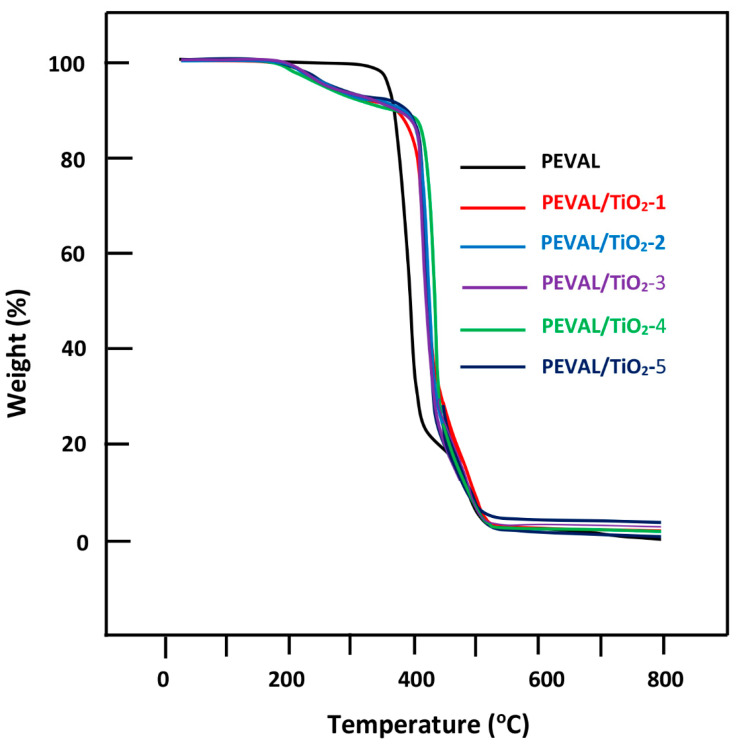
TGA thermograms of pure PEVAL and the PEVAL/TiO_2_ nanocomposites with different TiO_2_ contents.

The TGA thermograms of the PEVAL and PEVAL/TiO_2_ hybrid materials are gathered in Figure 4. As can be seen from these thermal curves, the decomposition of the virgin PEVAL took place in two main stages. The first decomposition, starting at 330 °C, involved the decomposition of greater part of the vinyl alcohol units, then the second at 410 °C led to the decomposition of the ethylene units. According to different authors [56,57], the thermal decomposition of poly(vinyl alcohol) (PVA) principally leads to the production of water and residual acetate groups, as well as quite a few chain-scission reactions resulting from radical decomposition reactions. According to Gomma et al. [58], the first zone of the thermal decomposition of this poly(vinylalcohol) (PVA), which is localized between 200 and 350 °C, is attributed to the loss of the water bond of the polymer chains. The second region between 340 and 450 °C is associated with the decomposition and carbonization of the polymer. On the other hand, Kumar and Sing [59] investigated the thermal degradation of poly(ethylene), and the main products that resulted were different volatile saturated and unsaturated hydrocarbons. The incorporation of TiO_2_ in a PEVAL matrix showed a non-negligible increase in the stability of this copolymer ranging between 30 and 35 °C depending on the composition of the material hybrid prepared, accompanied by a release of 6.5 to 8.2 wt% of water.

### 3.5. SEM Analysis

**Figure 5:**
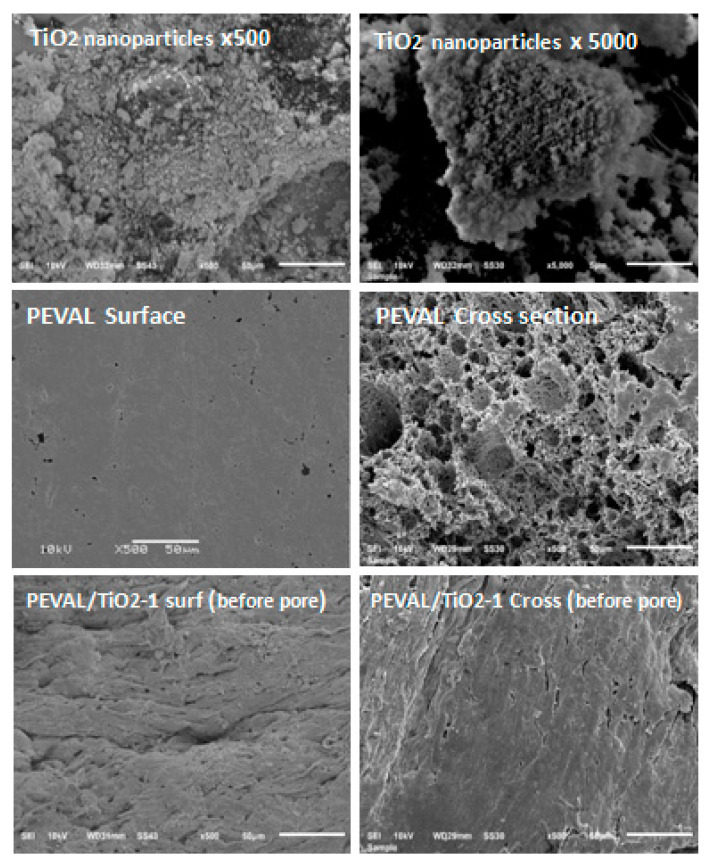
SEM images of TiO_2_ nanoparticles magnified 500 and 5000 times, showing the surface morphology and cross-section of pure PEVAL and a PEVAL/TiO_2_-1 nanocomposite film sample before pore connection.

Micrographs of the TiO_2_ nanoparticles, and the morphological surfaces of the unloaded PEVAL and the PEVAL loaded with 1 and 3 wt% of TiO_2_ as examples are presented in Figure 5. As seen in the TiO_2_ image, most of these particles appear to be gathered in aggregate forms of different sizes. This could be due to the presence of water molecules absorbed from moisture favoring the adhesion of TiO_2_ nanoparticles between them. The pure PEVAL shows a perfectly smooth morphology surface devoid of any relief that may interfere with those of the TiO_2_ particles when they are added. However, as shown in the bottom left, the incorporation of 1 wt% of TiO_2_ altered the surface morphology of the PEVAL, creating a relief resembling the surface of dough after being kneaded. On the other hand, the lower right image, which shows the surface morphology of the hybrid material containing 3% wt of TiO_2_, exhibits the uniform dispersion of these fillers, which are well covered by the copolymer. This could be due to the presence of good affinity between the two components of the hybrid material in which the PEVAL macromolecules chain by expanding in the solvent, which promotes the dislocation of the aggregated TiO_2_, leading to a uniform dispersion in the polymer matrix.

**Figure 6:**
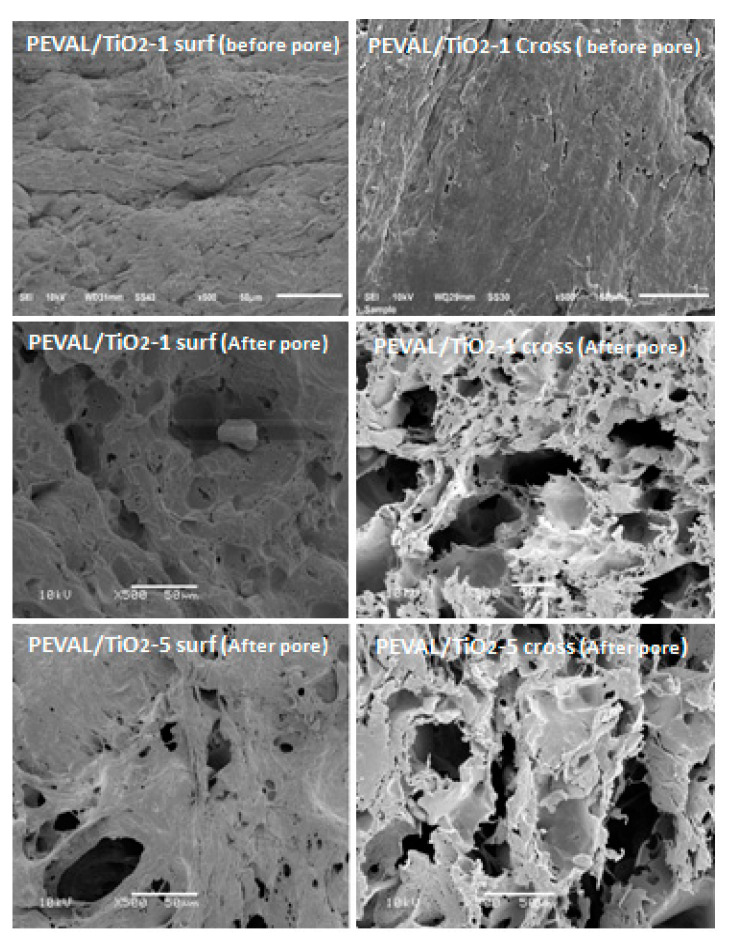
SEM micrographs of TiO_2_ nanoparticles, and the surface morphology and cross-section of virgin PEVAL and PEVAL/TiO_2_ nanocomposites containing 1, 3 and 5 wt% TiO_2_ after pore connection.

**Figure 7:**
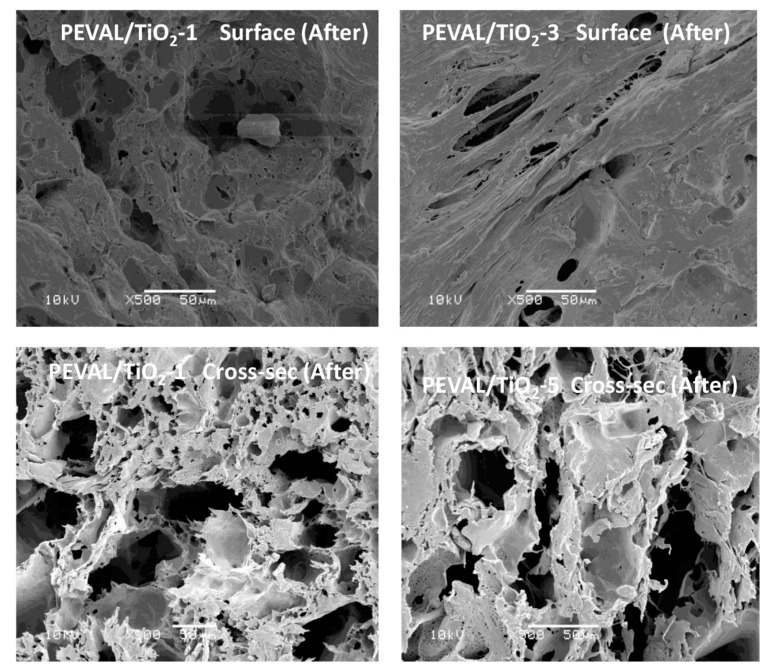
SEM micrographs of the surface morphology and cross-section of PEVAL/TiO_2_ nanocomposites containing 1 and 3 wt% TiO_2_ after removing Naph porogens.

Figure 6 shows photos of the surfaces and cross-sections of nanocomposite samples containing 1 and 3 wt% of TiO_2_ after porogen removal (Naph microparticles). These images clearly show the presence of pores on the surface of the material, which have almost circular shapes in the case of the sample containing 1 wt% of TiO_2_ and in the form of ellipses of variable size in the case of the one containing 3 wt% of this load. The photos at the bottom taken from the cross-sections of these samples clearly show the interconnection of these pores. Two types of pores are seen in the photo of the hybrid material containing 1 wt% of TiO_2_: those characterized by diameters varying between 5 and 20 μm and others that are relatively large (between 80 and 150 μm), thus reflecting the relief of the surface morphology of this sample. However, the sample that contained 3 wt% TiO_2_ was characterized only by a single pore type with the same diameter as those of the second type observed in the first sample, also reflecting its surface morphology. The small and dense pores observed in the PEVAL/TiO_2_-1 sample are also apparent in the same original material (i.e., even before the incorporation of the porogen), as shown in Figure 7, and the presence of a higher amount of TiO_2_ appears to have modified the texture of the original material, leading to a denser material and eliminating the pores formed by repulsions between the hydrophilic sequences of vinyl alcohol and the hydrophobic sequences of the ethylenic sequences of PEVAL.

### 3.6. Porosity and Pore Size Distribution

**Figure 8:**
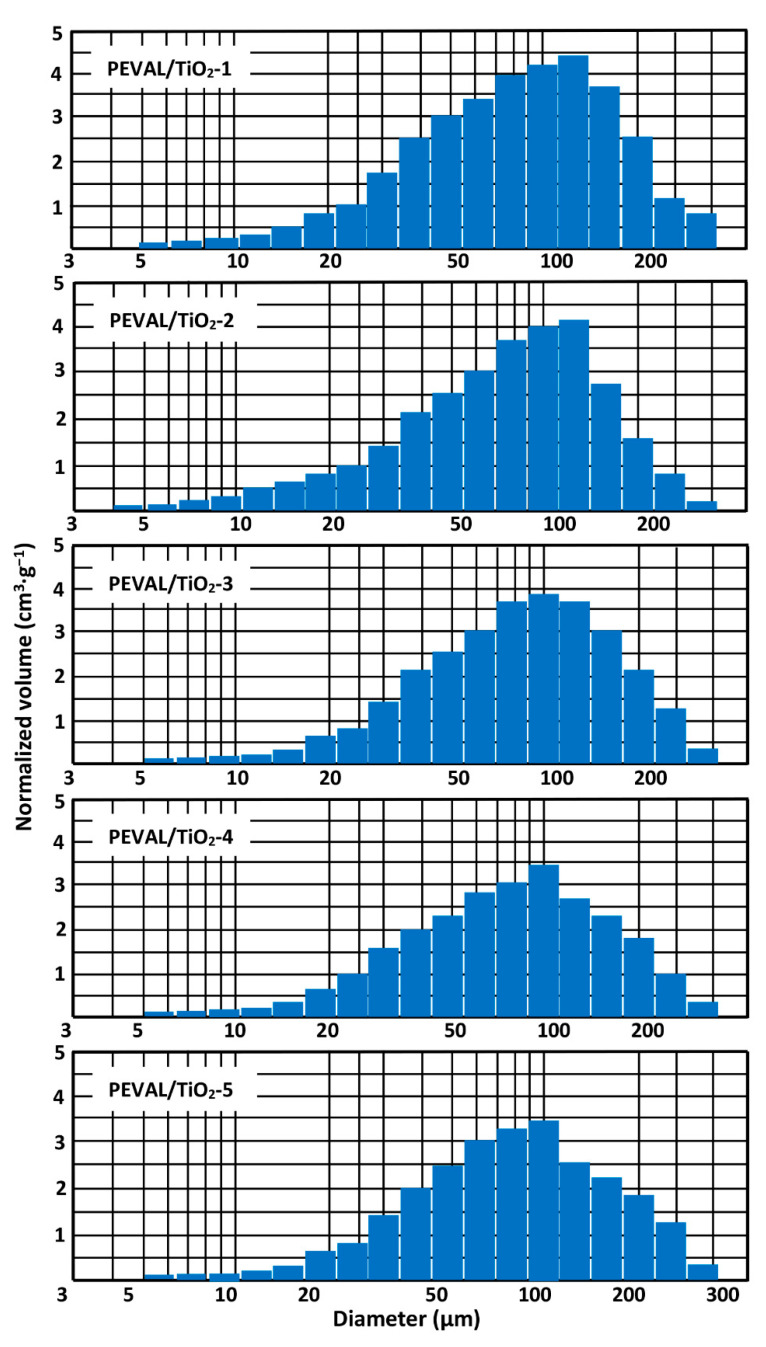
Pore size distributions in the microporous PEVAL/TiO_2_ hybrid materials with different TiO_2_ contents.

The results of the study of the porosity of the samples obtained by the pycnometer method are grouped in Table 3. These values indicate an average porosity of 82.76% for the sample containing 1% by weight of TiO_2_. This value decreased slightly with an increase in titanium oxide in the material. Similar results were also obtained using the poly(delta-valerolactone)/TiO_2_ system, which was attributed to the mechanical and thermal properties of this hybrid material [60]. Indeed, the decrease in the Tg value of the PEVAL with the increase in the TiO_2_ content in the composite observed by DSC analysis (from 62 to 53 °C) goes in the direction of increasing the flexibility of the resulting material. This promotes shrinkage of the pores under the action of the high vacuum created during the interconnection of the pores. As it can be seen from Figure 8, the pore size distribution in the PEVAL/TiO_2_ hybrid materials shows a wide pore size distribution, for which the maximum is between 30 and 120 μm for all compositions. As can be also observed in this figure, the presence of very small pores (<10 μm) can be a proof of the interconnection of the pores. The large distribution of pores sizes in these hybrid materials (4–200 μm), without a doubt, confirms the presence of a high percentage of interconnected pores. This confirms that the connection of the pores in these materials was indeed achieved by the disappearance of the walls separating the neighboring pores due to the high pressure exerted from the inside towards the outside of the pores subjected to the high vacuum during the porogen removal process.

### 3.7. Cellular Activity

**Figure 9:**
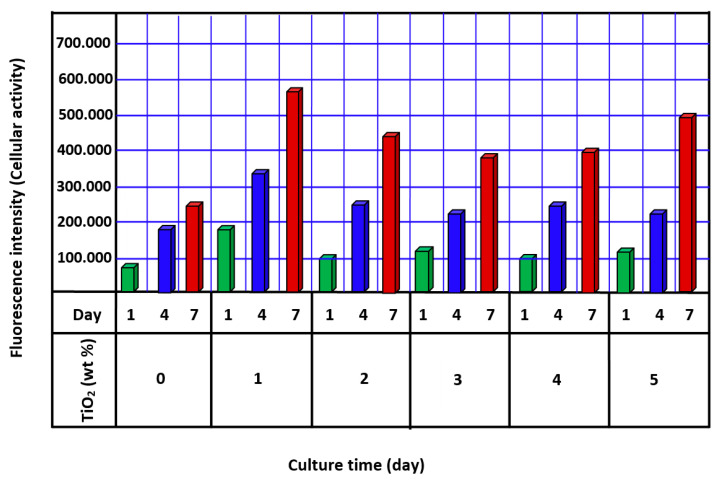
Cellular activity of HGFs cultured with different composite discs for 1, 4 and 7 days, after 1 h incubation in Alamar Blue^®^.

**Figure 10:**
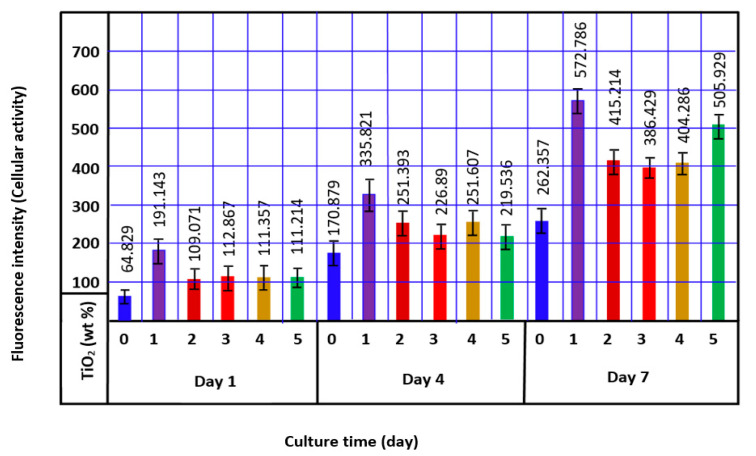
Cellular activity of HGFs cultured with different PEVAL/TiO_2_ scaffolds on Day 1, Day 4 and Day 7 after 1 h incubation in Alamar Blue^®^.

**Figure 11:**
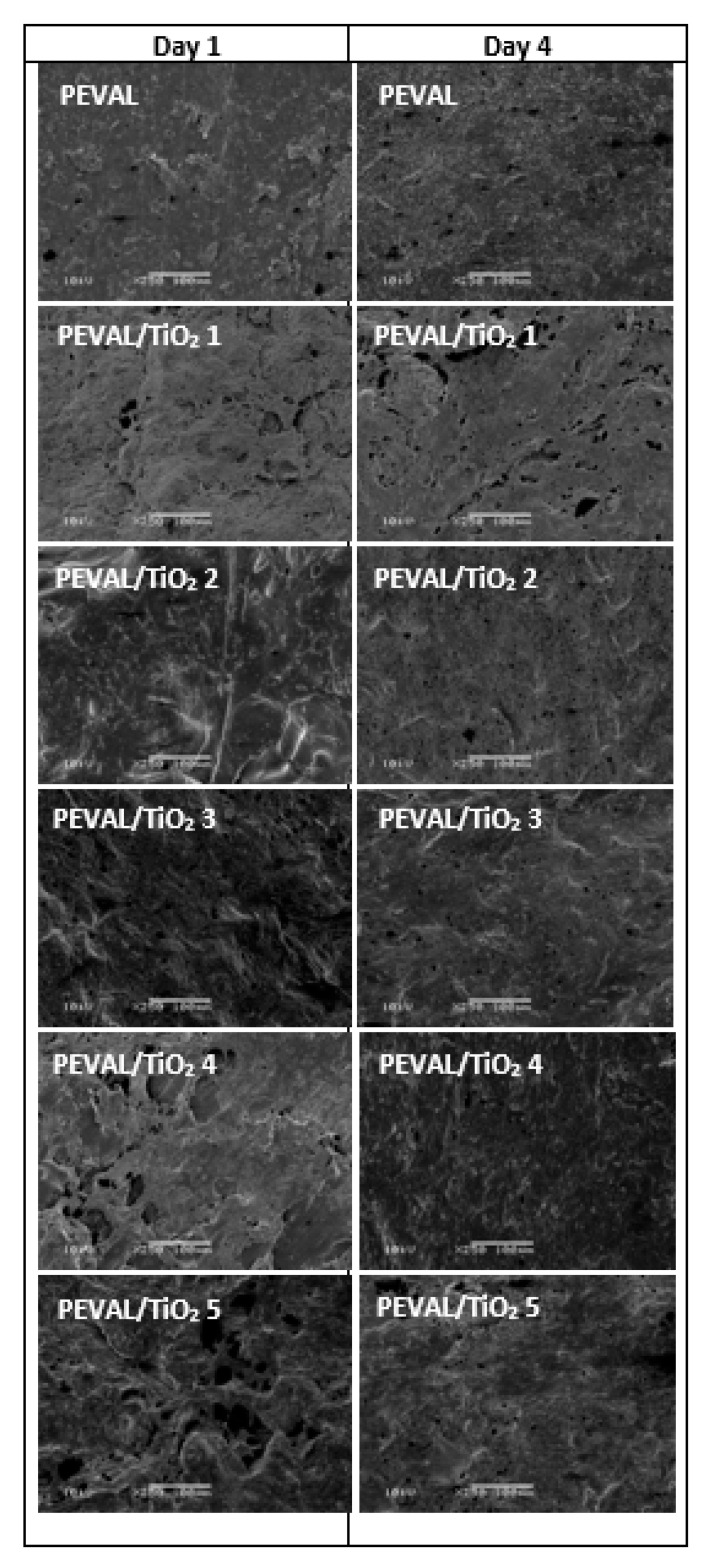
SEM images of HGFs cultured for 1 and 4 days.

HGFs showed increased cellular activity across the different culture time points. The cell activity increased in all groups from Day 1 to Day 7; this increase was statistically significant, as shown in Figure 9. Higher cell activity in all test groups (PEVAL/TiO_2_ scaffolds) was observed compared with the cellular activity of HGFs cultured with the control group (virgin PEVAL). On Day 1, HGFs cultured with PEVAL filled with 1 wt% TiO_2_ showed superior bioviability compared with HGFs cultured with virgin PEVAL and PEVAL containing 2, 3, 4 and 5 wt% TiO_2_. As shown in Figure 10, this difference is statistically significant. On Day 4, HGF was seeded in the PEVAL/TiO_2_ system; whatever its composition, it showed an increase in cell viability compared with the results observed on Day 1, and this increase was statistically significant (Figure 10). However, HGFs cultured on PEVAL/TiO_2_ scaffolds containing 2 and 4 wt% TiO_2_ showed significantly higher cell activity than the other groups (Figure 10). As shown in this figure, the same pattern was observed on Day 7: all tested groups showed significantly higher cell viability compared with the control group on Day 7 (*p* < 0.002). HGFs’ attachment to the scaffolds was investigated using SEM. The SEM images (Figure 11) showed that the HGFs were successfully cultured on all scaffold types. On Day 1, the HGFs had already formed a homogeneous surface on all scaffolds; however, SEM images of HGFs cultured on PEVAL/1wt%TiO_2_ showed a denser cell layer compared with the other membranes. The same was observed on Days 4 and 7 (data not shown).

## 4. Conclusions

The objectives of this work have been achieved. Indeed, PEVAL/TiO_2_ nanocomposites with different TiO_2_ contents were successfully prepared by the solvent casting method. The creation of interconnected and uniformly distributed pores in the prepared hybrid material was successfully achieved by using naphthalene microparticles as porogen. The pore size distribution in the PEVAL/TiO_2_ hybrid materials showed a wide pore size distribution, for which the maximum was between 30 and 120 μm for all compositions. The analyses by FTIR, DSC and XRD showed that the distribution of the nanoparticles in the PEVAL matrix was quite uniform. Thermal analysis of the hybrid material revealed that the incorporation of TiO_2_ nanoparticles into the PEVAL matrix resulted in a slight decrease in the T_g_ of the copolymer, keeping it always above body temperature (37 °C). The incorporation of TiO_2_ in the PEVAL matrix showed a non-negligible increase in the stability of this copolymer, ranging between 30 to 35 °C depending on the composition of the hybrid material prepared. The results of the cell activity tests on the virgin PEVAL and the PEVAL/TiO_2_ hybrid materials revealed an increase in cell activity across the different culture times. This cellular activity increased significantly in all groups from Day 1 to Day 7. Higher cellular activity in all test groups (PEVAL/TiO_2_ scaffolds) was observed compared with the cellular activity of HGFs cultured on the control (virgin PEVAL). HGFs’ attachment to the scaffolds investigated by the SEM method showed that the HGFs were successfully cultured on all scaffold types. On Day 1, the HGFs had already formed a homogeneous surface on all scaffolds; however, HGFs cultured on PEVAL/1wt% TiO_2_ showed a denser cell layer compared with the other membranes. The same was observed on Day 4 and Day 7.

## Figures and Tables

**Table 1:** Preparation conditions of PEVAL/TiO_2_ nanocomposites.

System	TiO_2_ (g)	PEVAL (g)	TiO_2_ (wt%)
PEVAL/TiO_2_-1	0.01	0.99	1.0
PEVAL/TiO_2_-2	0.02	0.98	2.0
PEVAL/TiO_2_-3	0.03	0.97	3.0
PEVAL/TiO_2_-4	0.04	0.96	4.0
PEVAL/TiO_2_-5	0.05	0.95	5.0

**Table 2:** Data deducted from the DSC thermograms of the PEVAL and PEVAL/TiO_2_ nanocomposites with different compositions.

System	T_g_ (°C)	T_m_ (°C)	∆H_m_ (J∙g^−1^)
PEVAL	60	183	66.78
PEVAL/TiO_2_-1	62	171	60.30
PEVAL/TiO_2_-2	61	171	47.73
PEVAL/TiO_2_-3	59	171	46.75
PEVAL/TiO_2_-4	57	171	45.08
PEVAL/TiO_2_-5	53	170	42.11

**Table 3:** The porosity of the PEVA/TiO_2_ nanocomposite with different TiO_2_ contents.

System	Porosity (%)
PEVAL/TiO_2_-1	80.2 ± 3.8
PEVAL/TiO_2_-2	78.3 ± 3.5
PEVAL/TiO_2_-3	74.7 ± 3.4
PEVAL/TiO_2_-4	72.8 ± 3.2
PEVAL/TiO_2_-5	72.8 ± 3.2

## Data Availability

Data that support the findings of this study are included in the article.
